# Powered air-purifying respirators used during the SARS-CoV-2 pandemic significantly reduce speech perception

**DOI:** 10.1186/s12995-021-00334-y

**Published:** 2021-09-30

**Authors:** Roxanne Weiss, Leon Guchlerner, Tobias Weissgerber, Natalie Filmann, Birgit Haake, Kai Zacharowski, Timo Wolf, Sabine Wicker, Volkhard A. J. Kempf, Sandra Ciesek, Timo Stöver, Marc Diensthuber

**Affiliations:** 1 Department of Otorhinolaryngology, Head and Neck Surgery, University Hospital Frankfurt, Goethe University, Theodor-Stern-Kai 7, 60590 Frankfurt/M, Germany; 2 Division of Audiological Acoustics, Department of Otorhinolaryngology, Head and Neck Surgery, University Hospital Frankfurt, Goethe University, Theodor-Stern-Kai 7, 60590 Frankfurt/M, Germany; 3 Institute of Biostatistics and Mathematical Modelling, Goethe University, Theodor-Stern-Kai 7, 60590 Frankfurt/M, Germany; 4 Department of Anesthesiology, Intensive Care Medicine & Pain Therapy, University Hospital Frankfurt, Goethe University, Theodor-Stern-Kai 7, 60590 Frankfurt/M, Germany; 5 Department of Internal Medicine II, Infectious Diseases, University Hospital Frankfurt, Goethe University, Theodor-Stern-Kai 7, 60590 Frankfurt/M, Germany; 6 Occupational Health Service, University Hospital Frankfurt Goethe University, Theodor-Stern-Kai 7, 60590 Frankfurt/M, Germany; 7 Institute for Medical Microbiology and Infection Control, University Hospital Frankfurt, Goethe University, Paul-Ehrlich-Str. 40, 60596 Frankfurt/M, Germany; 8 University Center of Competence for Infection Control of the State of Hesse, Goethe University, Paul-Ehrlich-Str. 40, 60596 Frankfurt/M, Germany; 9 Institute of Medical Virology, University Hospital Frankfurt, Goethe University, Paul-Ehrlich-Str. 40, 60596 Frankfurt/M, Germany; 10 German Centre for Infection Research, External partner site Frankfurt, Frankfurt/M, Germany; 11 Fraunhofer Institute for Molecular Biology and Applied Ecology (IME), Branch Translational Medicine and Pharmacology, Frankfurt, Germany

**Keywords:** COVID-19, SARS-CoV-2, Personal protective equipment, Powered air-purifying respirator, PAPR, FFP3 respirator

## Abstract

**Background:**

Due to the coronavirus disease 2019 (COVID-19) pandemic, interventions in the upper airways are considered high-risk procedures for otolaryngologists and their colleagues. The purpose of this study was to evaluate limitations in hearing and communication when using a powered air-purifying respirator (PAPR) system to protect against severe acute respiratory syndrome coronavirus type 2 (SARS-CoV-2) transmission and to assess the benefit of a headset.

**Methods:**

Acoustic properties of the PAPR system were measured using a head and torso simulator. Audiological tests (tone audiometry, Freiburg speech test, Oldenburg sentence test (OLSA)) were performed in normal-hearing subjects (*n* = 10) to assess hearing with PAPR. The audiological test setup also included simulation of conditions in which the target speaker used either a PAPR, a filtering face piece (FFP) 3 respirator, or a surgical face mask.

**Results:**

Audiological measurements revealed that sound insulation by the PAPR headtop and noise, generated by the blower-assisted respiratory protection system, resulted in significantly deteriorated hearing thresholds (4.0 ± 7.2 dB hearing level (HL) vs. 49.2 ± 11.0 dB HL, *p* < 0.001) and speech recognition scores in quiet (100.0 ± 0.0% vs. 2.5 ± 4.2%, *p* < 0.001; OLSA: 20.8 ± 1.8 dB vs. 61.0 ± 3.3 dB SPL, p < 0.001) when compared to hearing without PAPR. Hearing with PAPR was significantly improved when the subjects were equipped with an in-ear headset (p < 0.001). Sound attenuation by FFP3 respirators and surgical face masks had no clinically relevant impact on speech perception.

**Conclusions:**

The PAPR system evaluated here can be considered for high-risk procedures in SARS-CoV-2-positive patients, provided that hearing and communication of the surgical team are optimized by the additional use of a headset.

**Supplementary Information:**

The online version contains supplementary material available at 10.1186/s12995-021-00334-y.

## Introduction

A local outbreak of a previously unknown disease, COVID-19, caused by infection with a novel coronavirus, SARS-CoV-2 [1], in Wuhan, Hubai Province, China, rapidly developed into a global epidemic in early 2020 and is currently posing major challenges to the world’s healthcare systems.

This remarkably variable disease shows a broad spectrum of clinical manifestations, ranging from completely asymptomatic patients [2] to rapidly progressive courses with lethal outcome despite intensive care treatment [3]. According to current data, the main route of human-to-human transmission of the pathogen primarily occurs via respiratory droplets from infectious individuals or, less frequently, as a result of direct contact with SARS-CoV-2-contaminated surfaces [4].

The experience of the past year has shown that medical personnel in the operating room are exposed to a considerable risk of infection when treating SARS-CoV-2-positive patients [5]. Since high SARS-CoV-2 viral loads can be detected in the upper respiratory tract of COVID-19 patients [6], otolaryngologists, whose activities are focused on this area, are among the most exposed specialties. A particular high risk of virus transmission appears to emanate from aerosol-producing interventions, such as endonasal skull base surgery using high-speed drill [7] or tracheostomy [8, 9]. Adequate personal protective equipment (PPE) is required not only for operations on COVID-19 patients but also for emergency interventions, e.g. in case of acute respiratory distress or life-threatening bleeding in the upper airways. This is of particular importance when the SARS-CoV-2 test result is unknown or delayed.

For these high-risk interventions N95 respirators (which correspond to the European respirator standards FFP2/3) and goggles have been suggested to protect the surgeons [10]. However, there is some evidence that higher-level PPE in the form of powered air-purifying respirators (PAPRs) may be more effective in safely preventing infection of the surgeon, especially in aerosol-producing, high-risk procedures [11, 12]. As a consequence, the use of PAPRs is now included in some PPE recommendations for interventions during the COVID-19 pandemic [13–15].

In routine clinical practice, we have noticed that hearing and communication are significantly impaired when PAPRs are used. There is a small number of studies providing data which confirm this subjective impression [16, 17]. The purpose of this study was to provide an in-depth analysis of acoustic properties of a PAPR which could potentially lead to difficulties in communication. We also tested the impact on hearing and communication when two communication partners used PAPRs to simulate the situation between surgeon and assistant during surgery. The audiological data measured when using PAPRs was compared with those measured when wearing FFP3 respirators or surgical face masks. Moreover, we evaluated the utility of a headset when using a PAPR system as an aid to overcome audiological limitations.

## Material and methods

### Powered air-purifying respirator (PAPR)

**Fig. 1:**
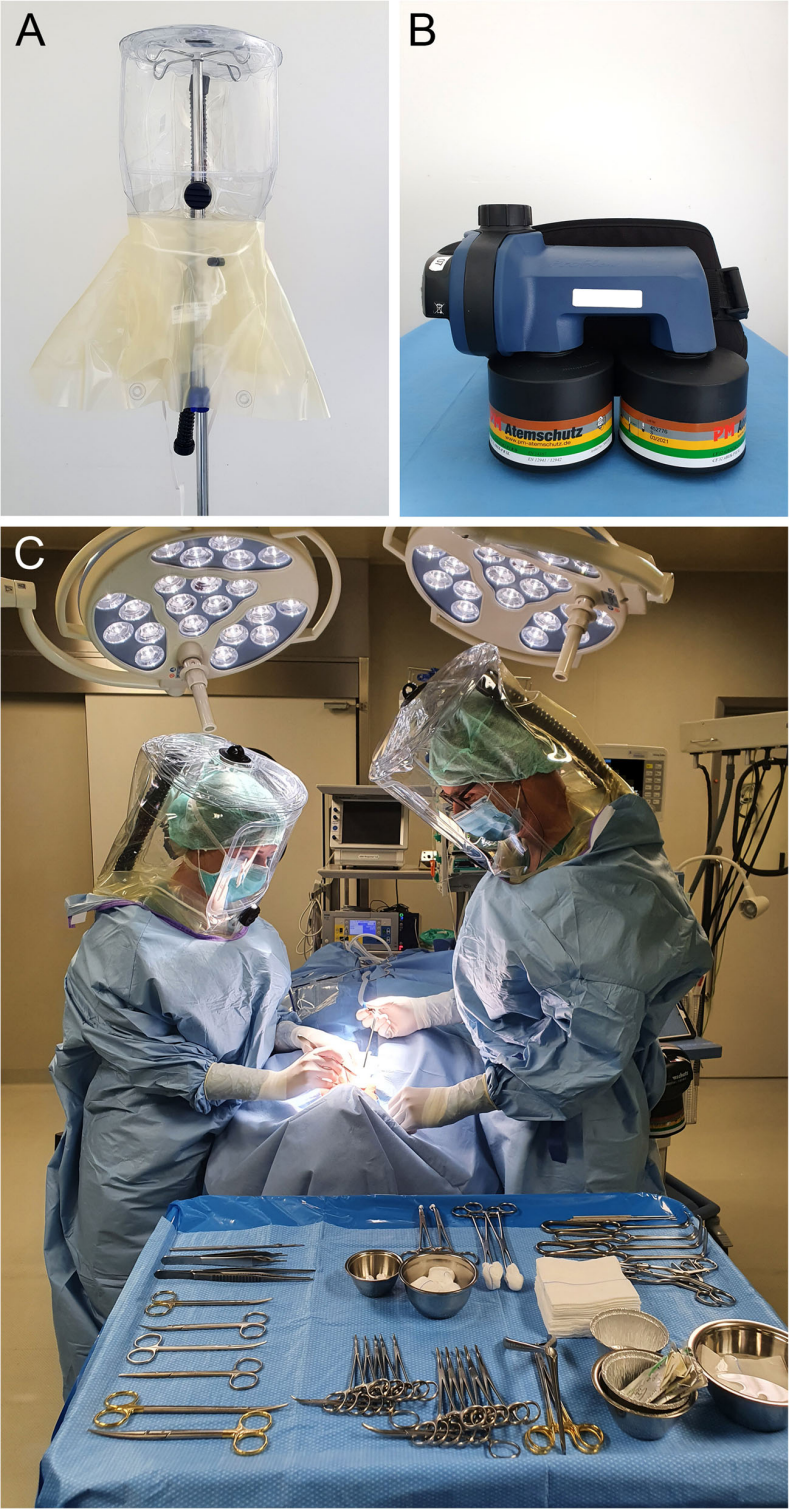
The powered air-purifying respirator (PAPR) composed of two main components, a translucent head top (**A**) and the blower-assisted respiratory protection system (**B**), represents an enhanced personal protective equipment (PPE) for use in high-risk surgical procedures (**C**)

The Tornado T9 respirator headtop (Scott Health and Safety Ltd., Skelmersdale, United Kingdom) (Fig. 1A) with connection to a respiratory blower is a CE-certified device that belongs to the category of PAPRs. The headtop completely encloses the head and is made of translucent polyurethrane, which provides all-round view. It was used in conjunction with a blower-assisted respiratory protection system (PM Proflow 2 SC, PM Atemschutz GmbH, Mönchengladbach, Germany) equipped with a CE-certified PM breathing protection filter CF 32 A2B2E2K2-P3 RD / CF 32 ABEK-P R SL (PM Atemschutz GmbH) suitable to protect against bacteria and viruses, gases and vapours as well as radioactive and highly toxic particles (Fig. 1B). Figure 1C shows the use of the PAPR in combination with an in-ear headset connected to a cordless telephone.

### Measurements of the acoustical properties of the PAPR system

**Fig. 2:**
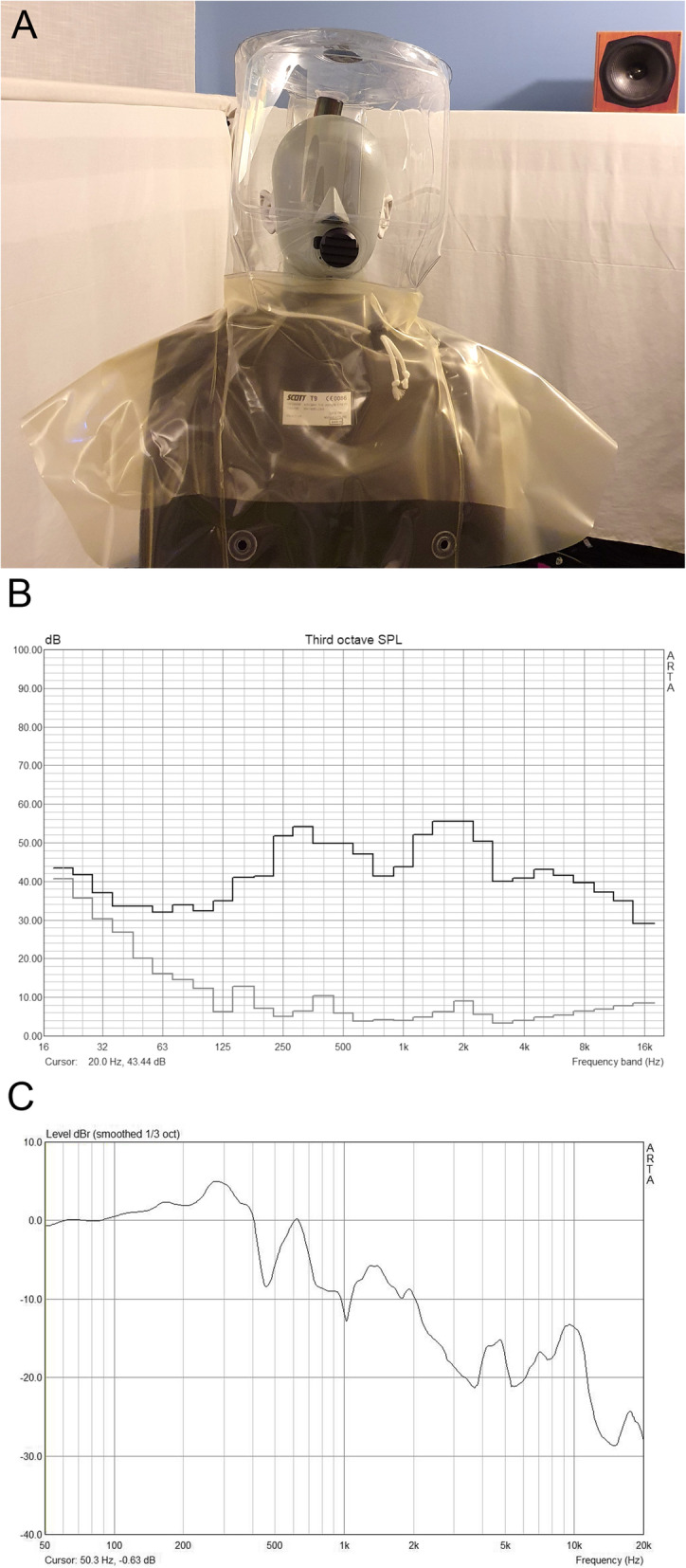
Measurement of the acoustical properties of the powered air-purifying respirator (PAPR) system. **A** Illustration of the PAPR system on a head and torso simulator used for measurements. **B** One-third octave band noise levels (dB SPL) of the PAPR system (black line) and the noise floor of the anechoic chamber (grey line). **C** Frequency spectrum of the sound insulation (dBr) of the PAPR calculated as difference between the transfer functions to the position of the left eardrum with and without PAPR system

Acoustical properties (sound attenuation and fan noise) of the PAPR system were measured in an anechoic chamber with the system worn on a head and torso simulator (Type 4100, Brüel & Kjær, Nærum, Denmark) connected to a measurement amplifier (Type Nexus, Brüel & Kjær) (Fig. 2A).

The noise level inside the headtop (at the position of the eardrum) generated by the fan of the blower-assisted respiratory protection system was measured in one-third-octave bands. To assess the sound insulation of the headtop, the transfer function from a loudspeaker (C5 tiny, KS Digital, Saarbrücken, Germany) to the left ear of the head and torso simulator was measured with and without the PAPR system. Analysis was conducted with ARTA software (ARTALABS, Kastel Luksic, Croatia).

### Participants and audiometric testing

Audiometric testing was performed in normal-hearing subjects (*n* = 10 health care professionals from our institution; *n* = 5 males, *n* = 5 females; age 28.6 ± 4.3 years) who were recruited to participate in the study on a voluntary basis. The participants had no previous regular experience in the use of PAPRs and received detailed instruction in the handling and use of this PPE before the audiometric measurements were performed. All tests were carried out in a sound-isolated booth with regularly calibrated audiometers and in free-field conditions with the PAPR system (headtop and activated respiratory protection system) and without the PAPR system. To overcome potential hearing deficits while wearing a PAPR, tests were additionally conducted in a simulated communication situation via wireless telephones. In this test condition, the subject used earphones (M2–022 earphone, M2-TEC, China) connected to a wireless telephone device (8242 DECT Handset, Alcatel-Lucent Enterprise, Colombes, France). A second wired microphone (M2–022 earphone, M2-TEC, China) was placed 10 cm (tone audiometry and Freiburg number and monosyllable tests) or 15 cm (speech reception thresholds, German matrix test) in front of the loudspeaker and connected to a second identical wireless telephone.

Warble tone hearing thresholds in free-field with and without PAPR system and with wireless telephone device were measured for the test frequencies 0.125/0.25/0.5/0.75/1/1.5/2/3/4/6/8 kHz. Speech perception scores in quiet were measured using the Freiburg number and monosyllable tests [18] at sound pressure levels of 65 dB SPL corresponding to a medium loud conversational level. Speech was presented from frontal direction at 0°.

Speech reception thresholds (SRTs, sound pressure level of speech with 50% correct word perception) in quiet were measured with the German matrix test (Oldenburg sentence test, OLSA) [19] in an adaptive procedure. Initial sound pressure level of the speech signal was 50 dB SPL. The test was performed in a closed-set mode. The OLSA test was performed without and with PAPR system (headtop and activated respiratory protection system). Additionally, the frequency characteristics of the target speaker were modified to simulate a real conversation, where the person speaking in the operating room would also be wearing PPE. This was realized by convoluting the speech signal of the OLSA test with the frequency response (i.e. dampening function) of three different types of PPE: 3-layer surgical face mask (KF-B P01, Kingfa Science &Tech, China), FFP3 respirator (REF 35100, FarStar Medical, Germany) and PAPR. The convolution was done with the software Equalizer APO (https://sourceforge.net/projects/equalizerapo/). The dampening functions of the 3-layer surgical face mask and the FFP3 respirator are shown in Supplemental Fig. S1. The experimental conditions of the audiometric tests are summarized in Tables 1 and 2.

**Table 1:** Audiometric evaluation in subjects using PAPR (manipulation of listener only)

Audiological test	Condition
	Listener (subject)
Warble tone audiometry	without PAPR
	with PAPR
	with PAPR and headset
Freiburg number and monosyllable test	without PAPR
	with PAPR
	with PAPR and headset
German matrix test (Oldenburg sentence test, OLSA)	without PAPR
	with PAPR
	with PAPR and headset

*PAPR* Powered air-purifying respirator

**Table 2:** Audiometric evaluation in subjects using PAPR, FFP3 respirator or surgical face mask with additional frequency response modification of the speaker (manipulation of listener and speaker)

Audiological test	Condition	
	Listener (subject)	Speaker
German matrix test (Oldenburg sentence test, OLSA)	SFM	SFM
	FFP3	FFP3
	PAPR	PAPR

*SFM* Surgical face mask, *FFP3* FFP3 respirator, *PAPR* Powered air-purifying respirator

### Statistics

Quantitative data are given as mean ± standard deviation (and median). Graphical presentation of the data was performed using GraphPad Prism 8 (GraphPad Software, San Diego, USA) and ARTA software (ARTALABS). Statistical data analysis was performed with R (version 4.0.4, R Foundation for Statistical Computing, Vienna, Austria) using linear mixed-effects models. Thereby, a maximum likelihood approach was used for fitting and the AIC (Akaike Information Criterion) was utilized for model selection of fixed and random effects with up to two-fold interactions as proposed by Seedorff et al. [20]. As all data arise from randomized block designs, where each block corresponds to one subject, subject-specific random effects were included to account for this correlation.

Audiometric data were analyzed using the following three models:

1. In order to analyze the effect of PAPR (and headset) on hearing thresholds, the conditions “without PAPR”, “with PAPR” and “with PAPR and headset”, frequency and the interaction between condition and frequency were included as fixed effects. The subject specific effect and the condition were included as random effects.
2. The speech recognition scores were analyzed including the condition (without PAPR, with PAPR, with PAPR and headset) as fixed effect and the subject specific effect as random effect.
3. To evaluate the influence of PPE on SRT the variable condition (without PAPR, with PAPR, with PAPR and headset, S: SFM / L: SFM, S: FFP3 / L: FFP3, S: PAPR / L: PAPR) was included as fixed and random effect and the subject specific effect as random effect. Hereby we considered the following two-step approach for the pairwise comparisons, as a difference of less than 2 dB is not clinically relevant: first, a test of equivalence for an equivalence margin of ±2 dB was carried out. Then, in case equivalence was not fulfilled, a test of difference was conducted.

For model building, the default “treatment contrasts” of the R language was used, which corresponds to dummy coding. Tukey’s all-pair comparisons were used for post-hoc-tests. Post-hoc *p*-values were adjusted with the single-step method. *P*-values ≤0.05 were considered statistically significant.

## Results

### Acoustical properties of the PAPR system

Sound pressure level of the noise generated by the fan of the blower-assisted respiratory protection system is shown in Fig. 2B (black line). The noise floor of the anechoic chamber is also shown (light grey line). One-third octave band noise levels ranged from 32 to 56 dB SPL with highest levels in the frequency region of 250–500 Hz and 1–2 kHz.

Sound insulation of the headtop is shown in Fig. 2C. For frequencies up to 400 Hz the headtop can be considered as acoustically transparent. For frequencies higher than 400 Hz an increased sound insulation could be observed with increasing frequency. In the frequency region of 3–5 kHz, which is important for the intelligibility of speech, sound insulation was in the range of 20 dB.

### Audiological data

#### Warble tone audiometry

**Fig. 3:**
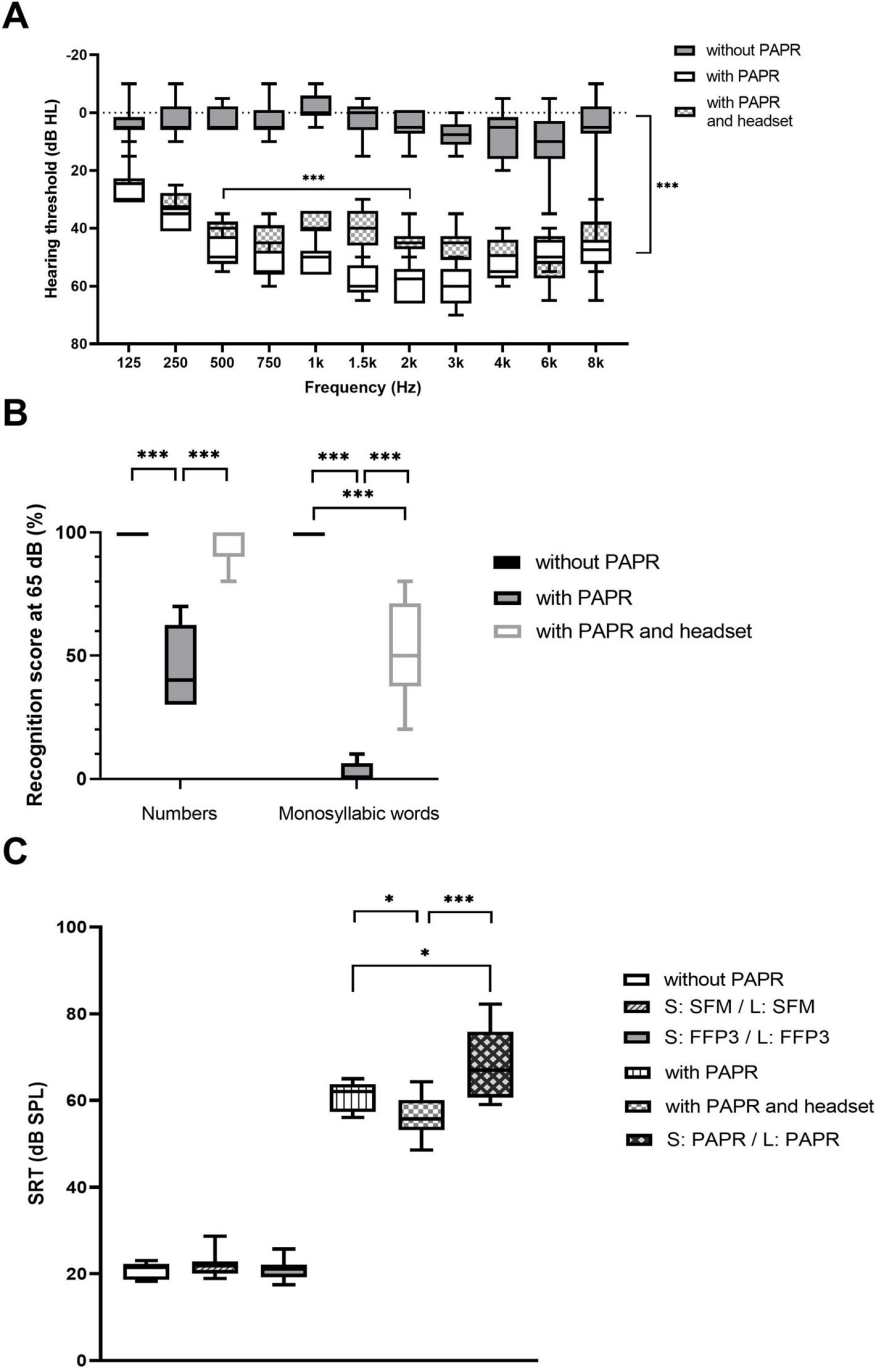
Impact of the powered air-purifying respirator (PAPR) on hearing performance in normal-hearing subjects (*n* = 10). **A** Boxplots of hearing thresholds (dB HL) with PAPR (white), without PAPR (grey) and with PAPR + headset (plaid) using tone audiometry. The vertical bar (***) applies for “without PAPR” vs. “with PAPR” and for “without PAPR” vs. “with PAPR and headset”. **B** Boxplots of speech recognition scores (%) for numbers and monosyllabic words (Freiburg speech test) without PAPR (black), with PAPR (grey) and with PAPR + headset (white) at 65 dB. **C** Boxplots of speech reception thresholds (SRTs) in dB SPL with PAPR (straight striped), without PAPR (white) and with PAPR and headset (plaid) as well as with attenuation of PAPR (oblique plaid), with attenuation of a surgical face mask (oblique striped) and with attenuation of an FFP3 respirator (grey) obtained with the German matrix test. Box plots show minima, maxima, interquartile range and median. Significant differences not indicated in the graph: without PAPR vs. with PAPR, without PAPR vs. with PAPR and headset, without PAPR vs. S: PAPR / L: PAPR, S: SFM / L: SFM vs. with PAPR, S: SFM / L: SFM vs. with PAPR and headset, S: SFM / L: SFM vs. S: PAPR / L: PAPR, S: FFP3 / L: FFP3 vs. with PAPR, S: FFP3 / L: FFP3 vs. with PAPR and headset, S: FFP3 / L: FFP3 vs. S: PAPR / L: PAPR (*p* < 0.001, respectively). S = speaker, L = listener, SFM = surgical face mask, FFP3 = FFP3 respirator. *p < 0.05; ***p < 0.001

Hearing thresholds in free-field audiometry without PAPR, with PAPR and with PAPR and headset are shown in Fig. 3A. Average free-field audiometry hearing thresholds for frequencies between 0.125 and 8 kHz without and with the PAPR system were 4.0 ± 7.2 dB HL vs. 49.2 ± 11.0 dB HL (median: 5.0 dB HL vs. 50.0 dB HL). A significant deterioration of the hearing thresholds caused by the sound attenuation of the head top and the fan noise of the activated respiratory protection system was detected (*p* < 0.001). When the PAPRs were used with a headset a significant improvement of the hearing thresholds was measured for the frequencies between 0.5 and 2 kHz (42.1 ± 5.4 dB HL, median 40.0 dB HL; *p* < 0.001).

#### Speech audiometry

Speech recognition scores without PAPR, with PAPR and with PAPR and headset are shown in Fig. 3B. Average number recognition score was 100 ± 0.0% (median: 100.0%) without PAPR at 65 dB SPL and significantly decreased to 45.0 ± 15.8% (median: 40.0%) at 65 dB SPL with the PAPR (*p* < 0.001). Likewise, the average monosyllabic word recognition score without the PAPR was 100.0 ± 0.0% (median: 100.0%) and was significantly reduced when the PAPR was used (2.5 ± 4.2%, median: 0.0%, *p* < 0.001). When using the PAPR system equipped with the in-ear headset, data showed a significant improvement of speech perception to 95.0 ± 7.1% (median: 100.0%, *p* < 0.001) for numbers and 52.5 ± 19.3% (median: 50.0%, *p* < 0.001) for monosyllabic words.

SRTs obtained with the OLSA for the test conditions “without PAPR”, “with PAPR”, “with PAPR and headset” and for the simulations of a talker using either surgical face mask, FFP3 respirator or PAPR are shown in Fig. 3C. Mean SRTs significantly increased (i.e. deteriorated) from 20.8 ± 1.8 dB SPL (median: 21.5 dB SPL) to 61.0 ± 3.3 dB SPL (median: 62.0 dB SPL) when the PAPR was used (*n* = 10, p < 0.001). When the PAPR was equipped with a headset, significantly lower SRTs were observed (56.2 ± 4.7 dB SPL, median: 55.8 dB SPL; *p* < 0.05).

OLSA results from measurements simulating the effects of different types of PPE used by the speaker showed that SRTs were not affected by the use of either a 3-layer surgical face mask or an FFP3 respirator, as for the comparisons of these conditions (“without PAPR vs. S: SFM / L: SFM”; “without PAPR” vs. “S: FFP3 / L: FFP3”; “S: SFM / L: SFM” vs. “S: FFP3 / L: FFP3”) the limits of the simultaneous 95%-confidence intervals for the differences were below 2 dB. Hence, it can be assumed that these conditions are equivalent.

When both speaker and listener (i.e. subject) used a PAPR (condition “S: PAPR / L: PAPR”), mean SRTs significantly deteriorated by 47.7 ± 10.0 dB SPL in comparison to the situation without PAPR (*p* < 0.001).

## Discussion

Since SARS-CoV-2 appears to be transmitted mainly via aerosol particles [21] and droplets [22], interventions in the upper airways, especially if they produce aerosols, are associated with a particularly high risk of SARS-CoV-2 infection for the surgeon as well as the surrounding team and may require enhanced PPE [23, 24]. Consequently, the most exposed specialties are otorhinolaryngology, anesthesiology and dentistry, which together account for 12% of COVID-19-related deaths among physicians [25]. Recent data show that at a university department of otorhinolaryngology in times of the COVID-19 pandemic, approximately 1 in 200 patients were found to be SARS-CoV-2-positive by polymerase chain reaction (PCR) testing [26]. In the field of otolaryngology, in particular the frequently performed routine interventions such as tracheostomy and surgical procedures with powered devices including functional endoscopic sinus surgery and mastoidectomy are considered high-risk procedures [13, 27].

For such interventions, the use of PAPRs has been proposed to ensure enhanced safety for the surgeons involved [13]. The successful use of PAPRs for viral infection control has already been reported in the management of severe acute respiratory syndrome (SARS) patients [28]. Our university hospital also has substantial experience in the use of PAPRs from the care and treatment of highly infectious Ebola patients [29]. Given the increasing incidences of new SARS-CoV-2 variants of concern in Europe [30] and limited availability of vaccines, but also the risk of further new respiratory infectious diseases with pandemic potential in the future, enhanced PPE, such as PAPRs, will continue to play a crucial role in protecting health care professionals from viral transmission.

The three-dimensional protection of the head and neck from splashes and aerosols and the superior filtering capacity of PAPRs compared to N95 respirators and surgical face masks are the defining qualities of PAPRs [11, 24]. Several drawbacks and limitations exist for N95 respirators such as suboptimal respirator fit due to facial hair [31] or significantly increased breathing resistance [32] which must be considered in case of long surgeries in infectious COVID-19 patients. In these cases, PAPRs can be a suitable and valuable alternative.

However, we found in clinical routine practice that hearing and thus communication is significantly compromised when the PAPR is worn and the respiratory protection system has been activated. This limitation is especially critical in case of emergency interventions, where rapid action and optimal communication are key.

To further explore these acoustic difficulties, we conducted hearing tests on normal-hearing subjects with and without PAPR. Tone audiometry revealed that the hearing thresholds were significantly deteriorated when the subjects wore the PAPRs with the blower-assisted respiratory protection system activated. Likewise, speech perception was significantly reduced under these conditions. This specific limitation of PAPRs has been reported previously [11, 16, 17] and has to be considered when this PPE is used in clinical routine. The underlying cause of these results became obvious when we characterized the acoustic properties of the PAPR. Firstly, the headtop of the PAPR causes substantial sound insulation. Secondly, the activated respiratory protection system produces noise with sound pressure levels up to 56 dB. Both factors together result in the detected restrictions in hearing and communication. Kempfle et al. [16] showed a mean deterioration of hearing thresholds of about 40 dB in a different PAPR system. In our study, the measured hearing thresholds were even 10 dB worse. The obtained mean reduction in word recognition scores of about 50% was comparable with the results reported in the study by Kempfle et al. [16]. Palmiero et al. [17] performed measurements of speech transmission index (STI) for different types of PPE and also reported a significantly lower (i.e. worse) STI for PAPR systems compared with N95 respirators and 3-layer surgical face masks.

To compensate for these limitations in hearing, the benefit of in-ear headsets was tested. In tone audiometry, a significant improvement of hearing thresholds of about 10 dB was found in the frequency range between 0.5 and 2 kHz. On the one hand, sound insulation of the PAPR in this frequency range was overcome by the use of a headset. On the other hand, hearing thresholds could not be further improved because of the high fan noise level between 0.25 and 2 kHz. For lower and higher frequencies, no benefit was found by using the headset. This can be explained by the limited transmission of frequencies lower than 500 Hz and higher than 4 kHz via the telephone. Our data (number and word recognition results in quiet) also clearly show that hearing with PAPR is significantly improved when an in-ear headset is used. With PAPR mean word recognition score was almost 0% and improved to about 50% at a conversational level of 65 dB SPL. Taking redundancy of conversational speech into account it could be assumed that a sentence recognition score of 100% is achievable by using a headset. However, listening effort in such a communication situation is still highly increased compared to a conversation using other types of PPE (FFP3 respirator, 3-layer surgical face mask).

This finding was also reflected in the SRT measurements. It was shown that using a PAPR led to a significant deterioration of SRT (40 dB) whereas no clinically relevant impact of FFP3 respirators and 3-layer surgical face masks on SRT was found. In a test condition in which listener and speaker used a PAPR, SRTs increased even more. In the test condition “with PAPR and headset” a significant improvement of 5 dB in SRTs was found. Given that the slope of the OLSA discrimination function is 11.3%/dB, this corresponds to an improvement in speech perception of 56.5%.

In the present study, no clinically relevant impact of surgical face masks or FFP3 respirators on SRT in quiet was found, although the measured sound attenuation in the frequency range between 2 and 4 kHz is up to 10 dB (Supplemental Fig. S1). Other studies also found no deterioration in speech perception for surgical face masks in either quiet [33] or for high signal-to-noise ratios [34]. A subjective decline in communication ability is frequently reported in clinical routine practice when using an FFP3 respirator in comparison to a 3-layer surgical face mask. However, only speech tests in quiet in a sound isolated booth were conducted. It could be hypothesized that differences in speech perception between FFP3 respirator and 3-layer surgical face mask are revealed by conducting a speech test in noise. The major differences in the dampening function between FFP3 respirator and 3-layer surgical face mask are present for frequencies higher than 5 kHz and thus could potentially reduce the recognition of sibilants in noise. Reduced speech perception in noise was shown by Brown et al. [33] for surgical face masks and by Toscano et al. [34] for N95 respirators (dampening function in the range of FFP3 respirator) in test conditions in noise with lower signal-to-noise ratio. In addition, our tests do not measure the potential negative effects of FFP3 respirators on articulation due to the tight fit on the jaw and the lips which could also affect communication ability.

## Conclusions

In summary, we present a PAPR system that appears suitable for use in aerosol-generating procedures. However, the use of such a PPE is accompanied by limitations in hearing and communication. If these difficulties are overcome by providing health care professionals with headsets, PAPRs represent a PPE with a very high level of protection, which can be recommended for high-risk interventions during the SARS-CoV-2 pandemic.

Since PAPR systems can be used not only to protect health care professionals from SARS-CoV-2 infection but also from various other highly contagious respiratory diseases, it can be anticipated that our findings will also be relevant for future pandemics.

## Supplementary Information

**Additional file 1: Supplemental Fig. S1.** Dampening function (i.e. frequency response) of the surgical face mask (light grey) and the FFP3 respirator (black).

## Data Availability

The datasets used and/or analysed during the current study are available from the corresponding author on reasonable request.

## Abbreviations

COVID-19: Coronavirus disease 2019
FFP: Filtering face piece
HL: Hearing level
OLSA: Oldenburg sentence test
PAPR: Powered air-purifying respirator
PCR: Polymerase chain reaction
PPE: Personal protective equipment
SARS: Severe acute respiratory syndrome
SARS-CoV-2: Severe acute respiratory syndrome coronavirus type 2
SPL: Sound pressure level
SRT: Speech reception threshold
STI: Speech transmission index
