# Supplementary figures and images for: Powered air-purifying respirators used during the SARS-CoV-2 pandemic significantly reduce speech perception
Source: J Occup Med Toxicol. 2021 Sep 30;16:43. doi: 10.1186/s12995-021-00334-y (PMC8481762; doi:10.1186/s12995-021-00334-y)

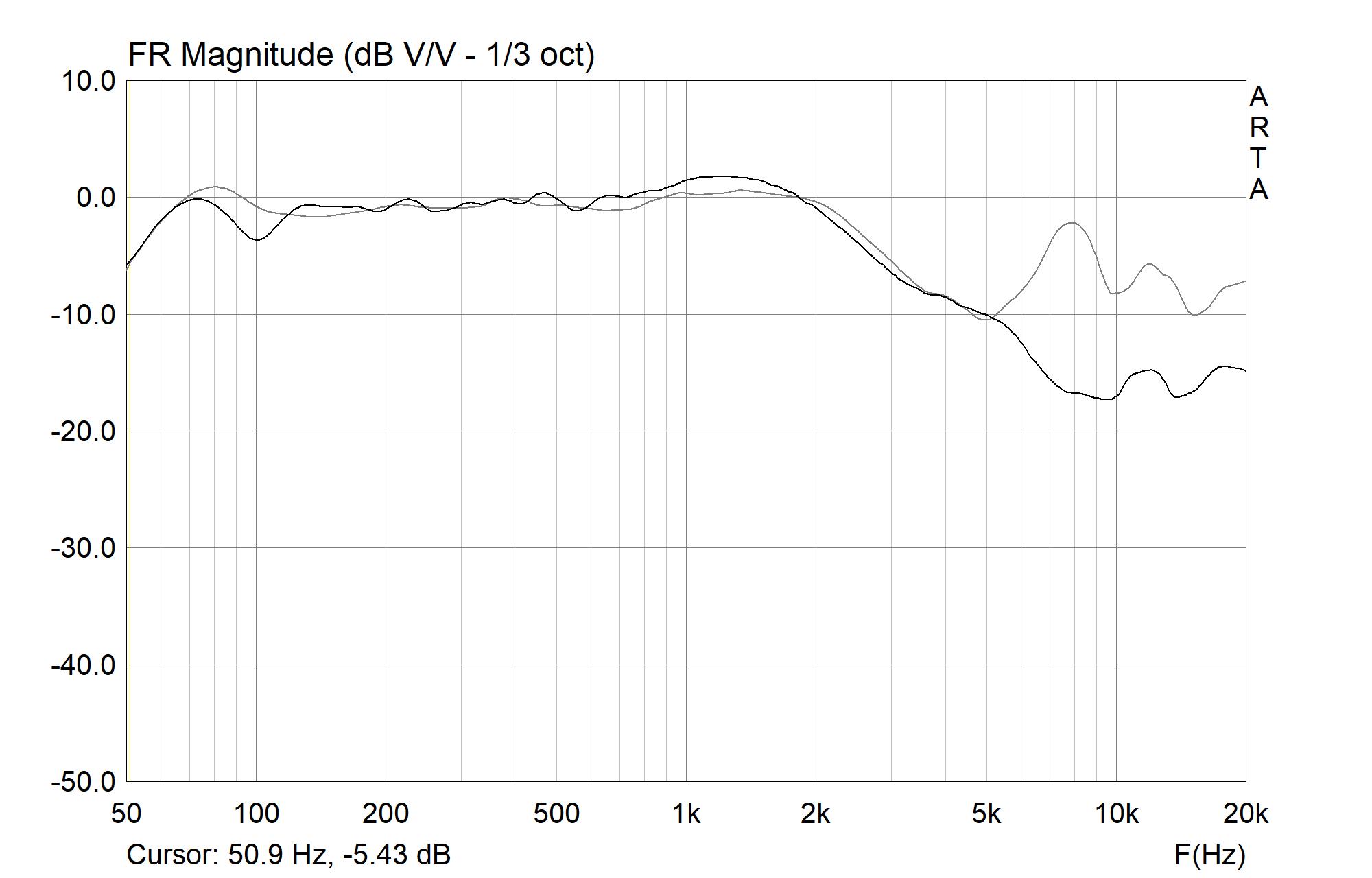

Supplement: Supplementary file 1 — Additional file 1: Supplemental Fig. S1. Dampening function (i.e. frequency response) of the surgical face mask (light grey) and the FFP3 respirator (black). [file 12995_2021_334_MOESM1_ESM.tif]
